# Comprehensive SUMO Proteomic Analyses Identify HIV Latency-Associated Proteins in Microglia

**DOI:** 10.3390/cells14030235

**Published:** 2025-02-06

**Authors:** Fergan Imbert, Dianne Langford

**Affiliations:** 1 Department of Neuroscience, Lewis Katz School of Medicine, Temple University, Philadelphia, PA 19140, USA; fergan.imbert@temple.edu; 2 Department of Cell Biology and Neuroscience, Rowan-Virtua School of Translational Biomedical Engineering and Sciences, Stratford, NJ 08084, USA; 3 Rowan-Virtua School of Osteopathic Medicine, Rowan University, Stratford, NJ 08084, USA

**Keywords:** SUMOylation, HIV, microglia, post-translational modifications, CNS

## Abstract

SUMOylation, the post-translational modification of proteins by small ubiquitin-like modifiers, plays a critical role in regulating various cellular processes, including innate immunity. This modification is essential for modulating immune responses and influencing signaling pathways that govern the activation and function of immune cells. Recent studies suggest that SUMOylation also contributes to the pathophysiology of central nervous system (CNS) viral infections, where it contributes to the host response and viral replication dynamics. Here, we explore the multifaceted role of SUMOylation in innate immune signaling and its implications for viral infections within the CNS. Notably, we present novel proteomic analyses aimed at elucidating the role of the small ubiquitin-related modifier (SUMO) in human immunodeficiency virus (HIV) latency in microglial cells. Our findings indicate that SUMOylation may regulate key proteins involved in maintaining viral latency, suggesting a potential mechanism by which HIV evades immune detection in the CNS. By integrating insights from proteomics with functional studies, we anticipate these findings to be the groundwork for future studies on HIV–host interactions and the mechanisms that underlie SUMOylation during latent and productive infection.

## 1. Introduction

In the central nervous system (CNS), viruses like human immunodeficiency virus (HIV) take advantage of the immunologically inert environment to persist in a latent state [1,2,3]. Viral integration, post-transcriptional mechanisms, and post-translational modifications (PTMs) have all been described as mechanisms that support the persistence of viral latency [4,5,6]. In recent years, the crucial role of post-translational modifications on the regulation of viral protein activity and its relation to viral latency have been explored [7,8]. PTMs are an essential mechanism used to dynamically regulate protein stability, localization, function, and signaling. PTMs include an array of modulations, including phosphorylation, acetylation, ubiquitination, and SUMOylation. These biochemical pathways are all highly regulated and have been linked to several neurodegenerative and inflammatory diseases [9,10,11,12].

In recent years, SUMOylation has gained increasing attention for its role in shaping host–pathogen interactions [13,14]. Small ubiquitin-related modifiers (SUMO, 18 kD) are members of the ubiquitin-like family of proteins that target lysine residues by covalent conjugation on a number of target proteins [15]. The SUMOylation pathway is most analogous to ubiquitination in that it involves an E1 (activating), E2 (conjugating), E3 enzymatic cascade. In mammals, five SUMO family members have been identified [16,17,18] with SUMO5 being reported in 2016. However, the expression of endogenous SUMO5 has yet to be confirmed, and it is possible that *SUMO5* is a SUMO pseudogene [17,19]. SUMO2 and SUMO3, the most abundant family members, share significant sequence similarity and are often referred to as SUMO2/3 [19]. Like SUMO5, SUMO4 is believed to be a SUMO pseudogene [18]. The unique functional capabilities, specificity, and expression profile of the SUMO paralogs provide the SUMOylation system with the ability to interact with several signaling pathways, regulating many essential processes.

HIV latency represents a major barrier to a functional HIV cure. Latent reservoirs, including those in resting CD4+ T cells and microglia, are shielded from immune surveillance and antiviral therapies, which target actively replicating viruses. The CNS is a key site for HIV reservoirs and is associated with neurological complications, including cognitive impairment and neuroinflammation, with microglia constituting the major viral reservoir and source of chronic inflammation. Understanding the mechanisms that govern HIV latency and reactivation in microglia is crucial for developing strategies to target these reservoirs. Recent studies have explored the role of the host SUMOylation system in HIV infection. SUMOylation has been shown to regulate the stability of transcription factors involved in HIV latency, including NF-κB and Sp1, which are crucial for maintaining a repressed state [20,21]. Microglia are productively infected by HIV and produce chemokines, cytokines, and other inflammatory factors, which may be major contributors to HIV-associated neurocognitive disorders (HAND), a spectrum of neurological disorders that range from mild cognitive impairment to dementia with a global prevalence of over 40% [22,23]. Unfortunately, the neuropathogenesis of HIV infection of the CNS is not completely understood and is more difficult to treat with combination anti-retroviral therapy (cART) than the periphery.

Microglia are a major component of the CNS innate immunity against viral infections, and studies have shown that they constitute a major reservoir for latent HIV infection [22,24]. Previous studies evaluated the molecular mechanisms that promote latency in microglia [25]. The nuclear cofactor chicken ovalbumin upstream promoter transcription factor-interacting protein 2 (CTIP2) inhibits HIV replication in microglial cells by recruiting a “viral latency complex” at the HIV viral promoter that consists of histone deacetylases (HDAC) transcription factors and the SUMO E3 ligase tripartite motif containing 28 (TRIM28). Several of the latency complex-associated proteins are known to interact with the host SUMOylation system. We speculated that the host SUMOylation system is induced during HIV infection, resulting in transcriptional silencing in microglia. Here, we show that SUMO conjugation is induced by proviral HIV infection, and that viral reactivation contributes to a significant loss of SUMO conjugation in a human microglia HIV latency model. Furthermore, using mass spectrometry analyses, we identified and quantified changes in host protein expression in microglia with latent infection and in microglia where the latent virus had been reactivated, uncovering distinct populations that reveal factors whose function is regulated by HIV. These data form the first proteomic and functional resource to understand nuclear SUMO response to HIV viral infection in the CNS.

## 2. Materials and Methods

### 2.1. Cell Culture

A novel human cell model of latently infected microglia (HC69) and the uninfected parent cell line (HC20) was provided by Dr. Alvarez-Carbonell [26,27]. HC69 cells were cultured in Dulbecco’s modified Eagle’s medium (DMEM; Thermo-Fisher, Waltham, MA, USA) supplemented with 1% fetal bovine serum (FBS) (Gibco, Waltham, MA, USA), and 1 uM dexamethasone (Sigma–Aldrich, St Louis, MO, USA). Dexamethasone is a glucocorticoid receptor (GR) agonist and mediator of anti-inflammation used to silence the HIV provirus in the HC69 cell model and has been previously reported [28]. While there are reports of dexamethasone inhibiting SUMOylation [29], for our studies, it was only used during initial culture stages to ensure low levels of GFP+ (HIV replication) cells in culture. Cells were washed and passaged in dexamethasone-free media for conducting final TNF-α, Mass Spec, TAK-981, and 2-D08 experiments. HC20 cells were cultured in DMEM with 10% FBS.

### 2.2. Confocal Microscopy and Immunofluorescence

All antibodies used are detailed below. Human frontal cortex tissue sections were treated with 1X antigen retrieval 6-buffer according to the Opal manual 4-color immunohistochemistry kit protocol (Akoya Biosciences, Marlborough, MA, USA). Tissue was blocked using the Opal blocking buffer (Akoya Biosciences). Multiplex immunohistochemistry (IHC) for SUMO 1, SUMO2/3, CTIP2, and TRIM28 labeling was performed according to Opal multiplex protocols. Target proteins were visualized using horseradish peroxidase-tagged fluorophore development with Opal dye 520, 540, 570, and 620. Slides were counterstained with DAPI (Akoya Biosciences). Slides were imaged via confocal imaging and were quantified using the Keyence BZ-X700 microscope, at 20X magnification of nonoverlapping images across the tissue section. These images were used to determine the average expression via fluorescence of each target substrate. 

### 2.3. Western Blot Analysis

Cells were lysed in RIPA lysis buffer (cat. no. 89900, Thermo Fisher Scientific, Waltham, MA, USA) supplemented with protease inhibitor (1 μg/mL final) (cat. no. 1861281, Rockford, IL, USA, Thermo Fisher Scientific, Waltham, MA, USA). Total protein concentration of the supernatant was measured by Pierce 660 nm Protein Assay (cat. no. 22660, Pierce, Thermo Fisher Scientific, Waltham, MA, USA) according to manufacturer’s protocol, and equal amounts (30 ug) of total proteins were denaturalized for 10 min at 96 °C in 2X Laemmli buffer (Sigma S340-1VL, St. Louis, MO, USA). Lysates were separated on a NuPAGE 4–12% gel. Proteins were transferred to a nitrocellulose membrane with the iBLOT2 (Invitrogen, Thermo Fisher Scientific, Waltham, MA, USA) machine at 25 V for 7 min. Proteins were analyzed by immunoblotting and visualized with enhanced chemiluminescence (Invitrogen, Thermo Fisher Scientific, Waltham, MA, USA). The relative intensity of individual bands, or gel lanes in the case of SUMO2/3 and SUMO1 blots, was analyzed by IMAGE J (Java 8, NIH, Bethesda, MD, USA). The protein bands from untreated cells were used as controls and were normalized to β-actin.

### 2.4. Reagents

The primary antibodies against SUMO2/3 (mouse monoclonal, ab81371; WB: 1:1000, IF 1:100), SUMO1 (rabbit recombinant monoclonal, ab32058; WB: 1:1000, IF: 1:100), UBA2 (rabbit recombinant monoclonal; WB: 1:2000, IF: 1:100), CTIP2 (rabbit polyclonal, ab28448; WB: 1:5000, IF: 1:100), and SENP2 (mouse monoclonal, ab131637, WB: 1:1000, IF: 1:100) are from Abcam, Cambridge, UK, as well as TRIM28 (rabbit polyclonal, PA5-27648, Thermo Scientific, Waltham, MA, USA; WB: 1:5000, IF: 1:100). WB, western blot; IF, immunofluorescence. The SUMO inhibitors TAK-981 and 2-D08 were obtained from MedChemExpress (Monmouth Junction, NJ, USA) and Cayman Chemical (Ann Arbor, MI, USA), respectively.

### 2.5. Flow Cytometry

For the cellular inhibition effects of TAK-981 and 2-D08 and viral reactivation studies, quantification of GFP expression was performed by flow cytometry analysis using Guava easyCyte system (EMD Millipore, Burlington, MA, USA), and the data were analyzed using InCyte v3.1. Prior to analysis of reactivation studies, HC69 cells were collected, washed in PBS, and immediately fixed in 4% paraformaldehyde in PBS. The effect of TAK-981 and 2-D08 was evaluated as previously described [30,31,32,33]. Briefly, Jurkat 3C9 and HC69 cells were treated with different concentrations of 2-D08 or TAK-981 for 1 h. TNF-α was then added to the cell culture, and GFP expression was measured 24 h of post-viral activation.

### 2.6. PPI Analysis

To evaluate the interactions between proteins and to identify HIV-1 latency-associated hub genes, PPIs were first evaluated using the STRING database (https://string-db.org/cgi/input.pl (accessed on 1 Novemebr 2024).

### 2.7. Statistical Analysis

Statistical analyses were performed using *t*-tests assuming unequal variances; *p*-values were calculated accordingly and indicated on all graphs. All data are presented as mean ± SEM (standard error of mean, n ≥ 3). Statistical significance was defined as * (*p* < 0.005), ** (*p* < 0.001), *** (*p* < 0.0001). All graphs were generated using GraphPad Prism software, Version 10.10 (264).

### 2.8. Illustrations

Illustration was created using the BioRender platform (https://www.biorender.com).

## 3. Results

### 3.1. Viral Reactivation in a Latent HIV Microglia Model Induces a Loss of Global SUMO2/3 Conjugation

**Figure 1:**
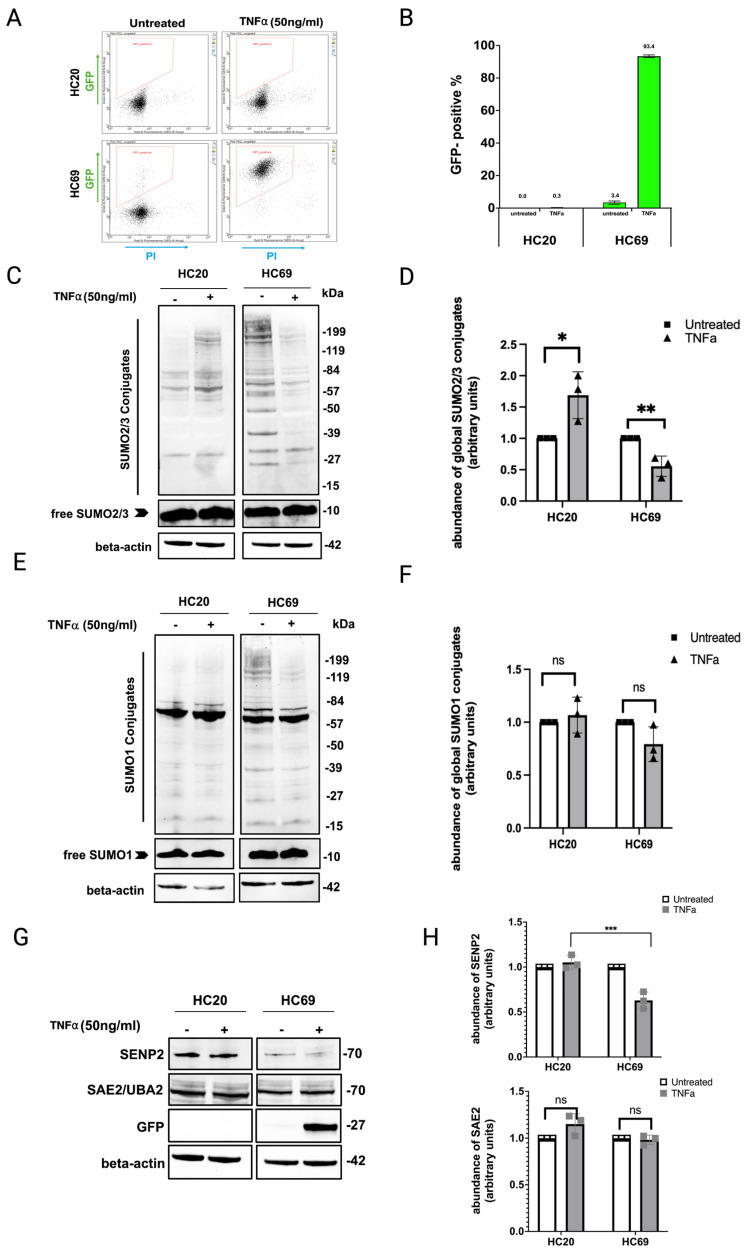
HIV-1 reactivation decreases the abundance of SUMO2/3 conjugates. (**A**,**B**) TNF-α treatment reactivates latent virus in microglia (HC69) as indicated by increased GFP expression. (**C**,**E**,**G**) Following activation of latent HIV in HC69 microglia, cells were lysed 24 h later, and global SUMOylation levels were assessed by Western blot with anti-SUMO1, -SUMO2/3, -SENP2, and -SAE2 antibodies. Representative blots are shown. Quantification of SUMO signals was normalized to beta-actin expression (loading control). (**D**,**F**,**H**) Data were presented as mean ± SD (n = 3), and asterisks denote statistical significance (*p*-values were calculated using *t*-test assuming unequal variance; * (*p* < 0.005), ** (*p* < 0.001), *** (*p* < 0.0001); ns, not significant). The parent cell line HC20 without HIV-1 provirus treated with TNF-α served as control.

Increased global cellular SUMOylation contributes to latency during viral infections [34]. To characterize the role of SUMOylation in HIV latency in microglia, we used an HIV-latently infected microglial cell line (HC69) and the uninfected parent cell line (HC20) as our models. The HC69 cell line is superinfected with VSV-g pseudo-typed HIV composed of a 3′ fragment of pNL4-3 (Dgag/pol), carrying a green fluorescent protein (GFP) reporter [27]. HIV activation of viral auxiliary proteins is essential for viral pathogenesis (Tat, Rev, Env, Vpu, and Nef) (Table 1), and GFP expression can be induced upon treatment with human tumor necrosis factor-alpha (TNF-α), a potent inducer of viral gene expression. As shown in Figure 1A,B, when treated with TNF-α, HC69 cells overwhelmingly express GFP, indicating viral replication. Western blot analysis of total HC69 cell lysates showed an increased abundance of proteins modified by both SUMO1 and SUMO2/3 (Figure 1C,D, and Figure 1E,F, respectively). Notably, the global levels of proteins SUMOylated by SUMO2/3 were significantly higher in HC69 cells compared to the uninfected human microglial cell line (HC20) (Figure 1C), whereas the amounts of free, unconjugated SUMO1 and SUMO2/3 were similar in both cell lines.

To determine the impact of viral reactivation on cellular SUMOylation in the HIV latent microglial model, we measured the expression of SUMO2/3 and SUMO1 in HC69 cells treated with TNF-α. As shown in Figure 1C, the global abundance of proteins modified by SUMO2/3 is significantly decreased, with a marginal decrease in the abundance of SUMO1 conjugation (Figure 1E). In the parent uninfected microglial cell line (HC20), western blot analysis revealed that TNF-α treatment triggers an increase in the abundance of proteins modified by SUMO2/3, but not SUMO1 (Figure 1C,E). This increase in protein SUMOylation levels is well documented in cellular stress conditions, including heat shock, hypoxia, and oxidative stress [35]. Furthermore, the significant increase in SUMO2/3 conjugation compared to SUMO1 is because SUMO2/3 exists mainly in its free, unconjugated form and is readily available to respond to certain stressors, whereas SUMO1 exists largely in its conjugated form [36]. These data suggest a specific deconjugation of SUMO2/3 in response to latent HIV reactivation in vitro.

To further characterize SUMO modulation following HIV viral reactivation in microglia, we focused on other proteins involved in the SUMOylation pathway. HIV can modulate the SUMOylation system by targeting SUMOylated host proteins for proteasomal degradation, or by targeting the SUMO E1, E2, or E3 enzymes. Alternatively, they can target SUMO-specific proteases (SENPs), which are responsible for SUMO deconjugation from target substrates. To assess these possibilities, we measured the protein expression of SAE2 (SUMO E1 subunit) and SENP2 in HC20 and HC69 cells in the presence and absence of TNF-α (Figure 1G,H). Western blot analyses revealed that SAE2 and SENP2 levels were not affected by treatment with TNF-α. However, levels of SENP2 were decreased in both the latently infected and TNF-α-stimulated HC69 cells. This decrease was statistically significant (*p* = 0.0003) when compared to the HIV latently infected HC69 cells to the uninfected, parent HC20 cells. Taken together, these findings suggest that treatment of latently infected cells with TNF-α induces SUMO2/3 deconjugation but does not affect levels of essential proteins in the SUMOylation pathway, which could be due to decreased expression of SUMO2/3 and/or its target substrates.

### 3.2. HIV Infection Decreases SUMO2/3 Protein Expression in Human Brain Tissue

**Figure 2:**
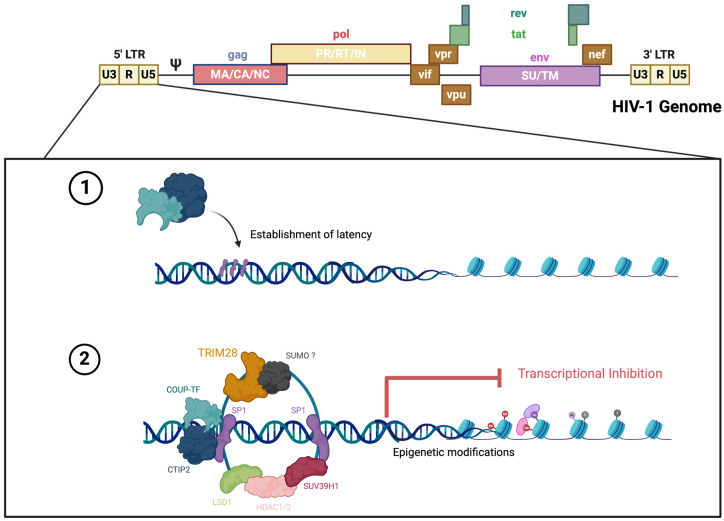
Model of the establishment of HIV-1 latency in microglia with CTIP2 as a central factor in the process. This figure was created using BioRender.

The establishment of HIV latency in microglia is mediated by CTIP2 through recruitment of a chromatin modifying complex (or viral latency complex) to the viral promoter in the 5′ long terminal repeat (LTR) (Figure 2). CTIP2 is anchored at Sp1 sites on the viral promoter and acts as a scaffold for the recruitment of latency-associated proteins, which consists of several chromatin-modifying proteins, including histone deacetylases 1 and 2, HDAC1 and HDAC2, SUV39H1 histone lysine methyltransferase, and lysine demethylase 1 (LSD1) [25]. Previous reports identified CTIP2 as a SUMO1 substrate in the brain [37]. Importantly, the SUMO E3 ligase, TRIM28, was reported to associate and cooperate with CTIP2 at the viral latency complex to silence HIV transcription [38]. Whether the SUMO paralogues are associated with the viral latency complex is unknown. To that end, we sought to evaluate the correlation between CTIP2, TRIM28, SUMO1, and SUMO2/3 expression and HIV infection in the human brain.

**Figure 3:**
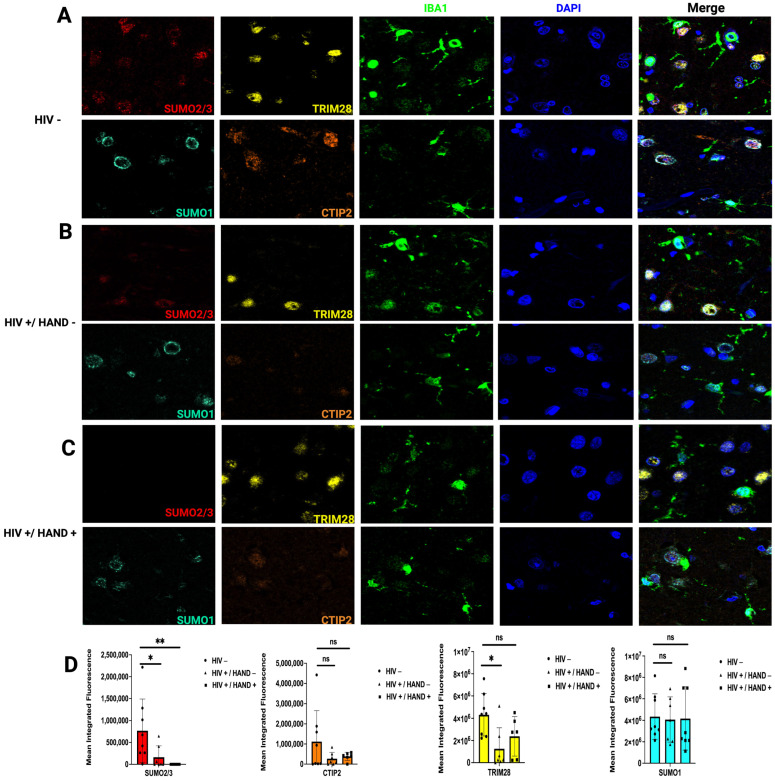
Analysis of HIV-1 latency-associated and SUMO paralogue protein expression in (**A**) HIV-negative controls, (**B**) HIV+/HAND-, and (**C**) HIV+/HAND+ brain tissues. Representative images of Opal multiplex immunohistochemistry targeting SUMO2/3 (red), SUMO1 (blue), CTIP2 (orange), and TRIM28 (yellow) from frontal cortex at 63× magnification are shown. IBA1 (green) was used as a lineage marker for microglia. (**D**) Target protein expression was quantified using the Keyence BZ-X700 microscope and batch analyses to determine average protein expression from 10 non-overlapping 20× images per brain section of each group. * (*p* < 0.005), ** (*p* < 0.001), ns, not significant.

To assess changes in SUMO and latency-associated proteins at the cellular level, we conducted multiplex immunofluorescence on brain tissue sections of the frontal cortex of people with HIV (PWH) compared to uninfected age-matched controls (Figure 3). An additional marker, ionized calcium-binding adaptor molecule1 (IBA1), was used to label microglia. In control individuals, we observed a robust expression of SUMO2/3 (Figure 3A) in both IBA1-posiive and IBA1-negative cells. In frontal cortex tissue of PWH without HIV-associated neurocognitive disorder (HIV+/HAND-) (Figure 3B) and PWH with associated neurocognitive disorders (HIV+/HAND+) (Figure 3C), we observed a significant loss of SUMO2/3 expression. Conversely, no changes in CTIP2 or SUMO1 expression levels were observed when comparing HIV-positive individuals, to HIV-negative controls (Figure 3A–C). TRIM28 expression was decreased in HIV+/HAND- individuals (Figure 3B), compared to HIV- individuals (Figure 3A). However, this difference became smaller in HIV+/HAND+ individuals (Figure 3C). Of note, the nuclear ring staining observed in SUMO1 in both HIV- (Figure 3A) and HIV+/HAND- (Figure 3B) was lost, with more diffuse nuclear staining in HIV+/HAND+ individuals (Figure 3C). Our results indicate that the SUMO2/3 paralogue is significantly reduced in PWH, confirming that decreased levels of SUMO2/3 conjugation in reactivated HIV latent microglia model may be associated with a loss of the SUMO2/3 substrate.

### 3.3. Quantitative Proteomics of HIV-Induced SUMO2/3 Deconjugation

**Figure 4:**
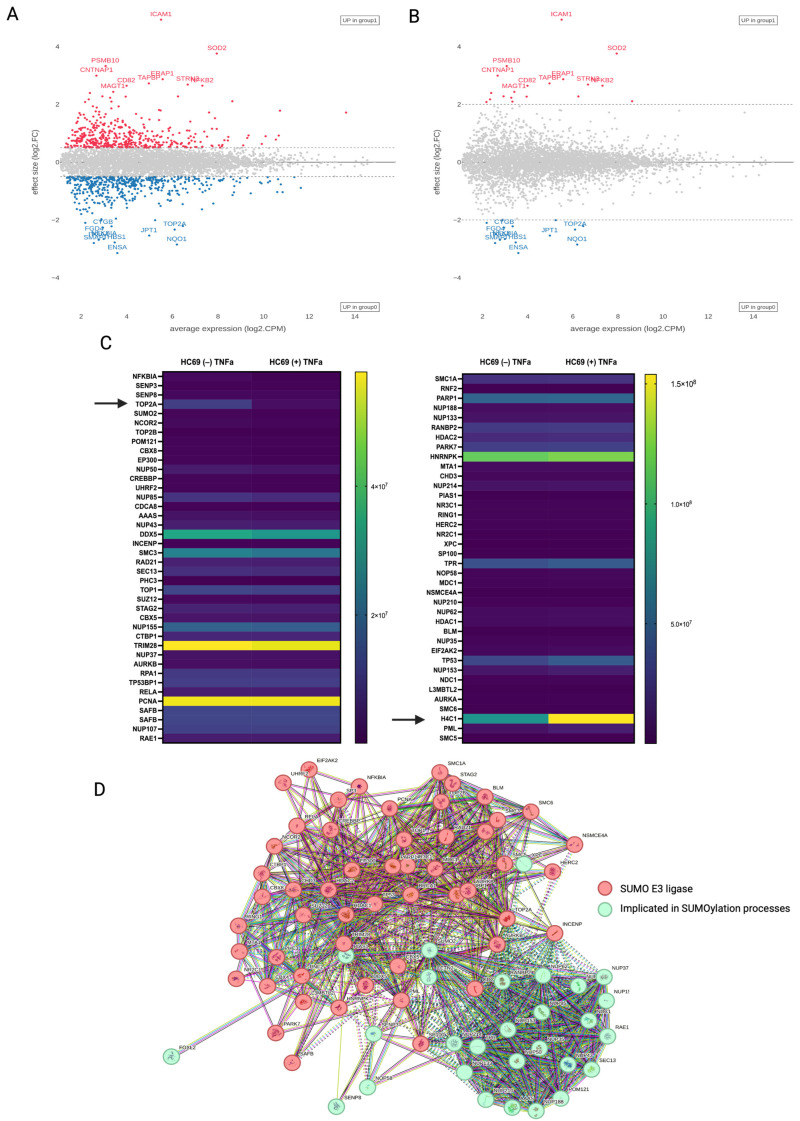
Quantitative proteomics of unstimulated and TNF-α-stimulated HC69 cells. (**A**,**B**) Bland–Altman plots of log2-fold changes in protein expression following HIV-1 reactivation in HC69 cells. Example proteins that increase or decrease in unstimulated and stimulated HC69 cells at a false discovery rate of 0.5 (**A**) and 2 (**B**) are labeled. See also Table S3. (**C**) Heatmap summary of proteins identified as SUMO “interactors” and associated fold change in stimulated HC69 cells as compared to unstimulated control cells. (**D**) Protein–protein association analysis (STRING) between all SUMO “interactors” was identified in the proteomics dataset, where nodes that share the same cluster ID are typically close to each other: cluster 1 (red) is SUMO E3 ligases; cluster 2 (green) is involved in SUMOylation, cellular transport, and the nuclear pore complex.

To gain an unbiased overview of the cellular and viral protein dynamics during HIV infection, we used the liquid chromatography with tandem mass-spectrometry (LC-MS/MS) proteomics strategy to identify changes in protein expression, with a focus on SUMOylation, in response to viral reactivation in the HC69 cell line. Using a false discovery threshold of 1%, we identified and quantified 5073 substrates (Figure 4A). Gene ontology (GO) analysis using the Enrichr [39,40] platform revealed a cellular component enrichment for intracellular membrane-bounded organelles (nuclear pore complex), nucleus, nucleolus, and cell-substrate junctions (Table S1), as well as molecular function enrichment for RNA, mRNA, and ATP binding and ubiquitin-protein ligase activity (Table S2). Interestingly, <1% of total identified substrates varied in total abundance greater than 2-fold after HIV reactivation in the latent HC69 microglia cell line (Figure 4B). GO analysis revealed a molecular function enrichment for protein phosphatase binding, nuclear factor kappa-light-chain-enhancer of activated B cell (NF-κB) binding, small GTPase binding, and nicotinamide adenine dinucleotide (NADH) dehydrogenase and protein homodimerization activity (Table 2). Several host proteins, including, CD82, intracellular adhesion molecule 1 (ICAM1), superoxide dismutase 2 (SOD2), alpha-endosulfine (ENSA), and NF-κB, have been implicated in HIV infection: HIV Tat stimulates the NF-κB-signaling pathways to enhance viral transcription [41]; SOD2 was reported to be upregulated in individuals with HIV encephalitis (HIVE) and in the simian immunodeficiency virus (SIV)-infected macaque model [42]; both cell surface and circulating ICAM1 are upregulated in HIV infection, but HIV Vpu counteracts host responses by inducing its proteasomal degradation [43,44]; reduced expression of the tetraspanin CD82 has been observed in T-lymphocytes in patients with HIV, with Vpu implicated in its downregulation [45]; and ENSA has been identified as differentially expressed in primary cell models of HIV latency [46,47].

Comparison of the quantitative changes from our proteomics data of SUMO proteins in response to latent HIV reactivation in microglia illustrated that SUMO1 and SUMO2 had decreased expression, while changes in the E1-activating enzymes (SAE1/SAE2) and SUMO proteases (SENP8; SENP3) showed no gross differences between unstimulated and reactivated populations. Thus, the overall decrease in SUMO conjugation in western analyses (Figure 1C,D) suggests that HIV-induced deSUMOylation is a result of a loss of the SUMO paralogues. Analysis of the purified samples identified showed that 84 (1.65%) substrates are known SUMOylation interactors. Comparison of the quantitative changes of known SUMO interactors in response to latent HIV-1 reactivation showed no major differences in protein expression, although histone 4 (H4C1) and topoisomerase 2 alpha (TOP2A) were differentially expressed (Figure 4C). It was previously reported that H4C1 stimulates HIV integration through direct interaction with the carboxy-terminal domain of integrase (IN) [48]. Additionally, SUMO modification of H4C1 by SUMO1 or SUMO2/3 is linked to transcriptional repression [49]. Human topoisomerases are highly regulated by post-translational modifications [50,51]. SUMOylation of topoisomerase II alpha (TOP2α) by SUMO1 or SUMO2/3 facilitates subcellular transport and protein–protein interactions, respectively [51,52,53]. TOP2α has previously been identified as differentially expressed in a cohort of individuals with acute HIV infection [54]. While the relative protein expression of topoisomerase I (TOP1) was not different between the unstimulated and HIV-reactivated populations, TOP1 was recently identified as a repressor of HIV LTR promoter activity [55]. Importantly, TOP1 and TOP2α associate and cooperate to regulate transcription [56].

Further analysis of our proteomic dataset revealed three members of the human SMC5/6 complex (SMC5, SMC6, and the SUMO ligase NSMCE4) to be enriched in both the latent and reactivated HC69 cell line, with increased expression in the reactivated population. The complex is required for telomere maintenance and mediates SUMOylation of the shelterin complex, a complex that protects telomeres from DNA damage and regulates telomerase activity [57]. Previous reports indicated that HIV-1 induces telomerase activity in monocyte-derived macrophages [58]. However, an exact mechanism for HIV-mediated telomerase activity in macrophages was not described. Here, we present the SMC5/6/SUMO ligase complex as a potential mechanism of viral-induced telomerase activity that could contribute to viral persistence and the establishment of macrophages as viral reservoirs. This hypothesis is supported by a study that identified that epigenetic silencing is mediated by SUMOylation of proviral and unintegrated HIV DNA by the SMC5/6 complex in primary T cells [59].

Additionally, a number of HIV proteins are known to be SUMOylated, and recent studies have demonstrated that HIV expression in HEK293 T-cells and in HeLa cells causes a reduction in host SUMOylation by targeting a subunit of the SUMO E1-activating enzyme [60,61,62,63]. Several of the proteins identified in our proteomic dataset have been implicated in the host SUMOylation system (Figure 4D). The degree of overlap with other studies and our proteomics data supports a correlation between the host SUMOylation system during HIV latency in microglia and a rationale for further studies investigating the functional consequences of protein SUMOylation during latent and productive HIV-1 infection.

### 3.4. Inhibiting SUMOylation Blocks TNF-α-Induced HIV Reactivation in Latently Infected Cells

**Figure 5:**
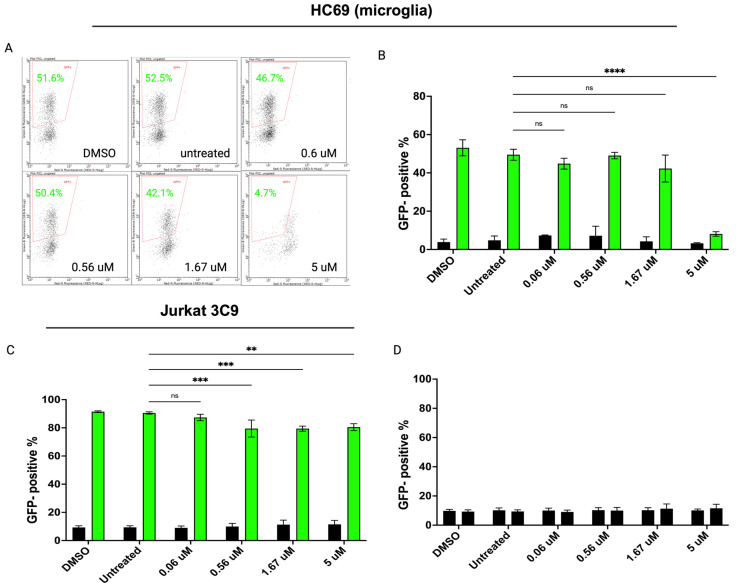
Inhibition of SUMOylation reduces HIV latency reactivation in vitro. (**A**) Representative flow cytometry profiles. HC69 (**B**) or Jurkat 3C9 (**C**) cells were treated with varying concentrations of TAK-981 for 1 h. GFP percentage was measured by FC before cells were then cultured either with diluent dimethyl sulfoxide (DMSO) or TNF-α. One day later, green fluorescent protein (GFP) expression was analyzed by flow cytometry. (**D**) The SUMO inhibitor, TAK981, does not reverse latency alone. GFP expression was measured before and after 24 h incubation of TAK981 at varying concentrations. Data shown are the mean ± SD of three biological replicates (** *p* < 0.01, *** *p* < 0.001, ns, not significant) (**** *p* < 0.0001, two-way ANOVA, Tukey’s test).

To determine if chromatin SUMOylation is crucial for the epigenetic silencing of proviral HIV DNA, we took advantage of the SUMO-specific inhibitor, (subasumstat 981) TAK-981 [64]. TAK-981 is an anti-cancer drug that is a selective inhibitor of a subunit of the SUMO-activating enzyme and blocks SUMOylation of target substrates. A dose-response experiment of TAK-981 at 0.6, 0.56, 1.67, or 5 μM for 1 h, followed by TNF-α treatment to induce HIV transcription, was performed in both human Jurkat 3C9 HIV and human microglial HC69 HIV latency models. The Jurkat 3C9 cell line is another model of HIV latency containing an integrated copy of GFP-HIV that can be reactivated by TNF-α [65]. After 24 h, cells were fixed and GFP was measured by flow cytometry (Figure 5A). We saw no significant effect on the percentage of GFP+ HC69 cells following the addition of TAK-981 compared to cells treated with TNF-α only or DMSO until high concentrations (Figure 5B). In contrast, TAK-981 significantly reduced the percentage of GFP+ Jurkat 3C9 cells in a dose-dependent manner (Figure 5C). We also confirmed that TAK-981 does not function as a latency-reversing agent (LRA) in HC69 cells (Figure 5D). Overall, the addition of TAK-981 prior to stimulation with TNF-α decreased the number of productively infected GFP+ cells in the latent Jurkat model, with little effect on the microglial model. These experiments demonstrate the inhibition of SUMOylation resulted in a decreased ability of TNF-α to reactivate HIV in the Jurkat model while having no significant impact on the microglial model, suggesting that microglia have unique reactivation mechanisms that do not rely on SUMOylation compared to T cells.

**Figure 6:**
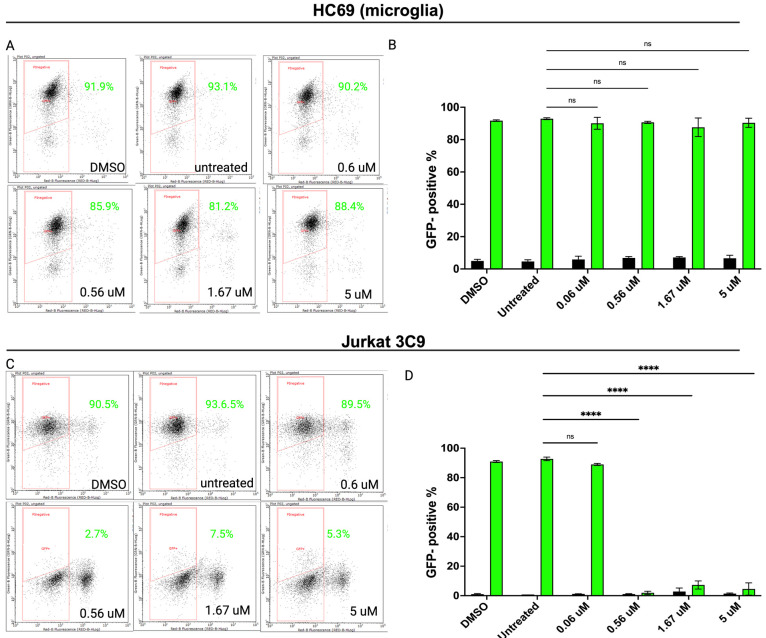
2-D08-inhibited HIV reactivation in latently infected cells. Dose-dependent reduction of GFP+ cells (HIV reactivation) in HC69 (**A**) and Jurkat 3C9 (**C**) cells with varying concentrations of 2-D08 compared to DMSO or control (without 2-D08). (**B**) and (**D**) are graphic representation of flow cytometry data from HC69 and 3C9 cells, respectively. All experiments were performed in triplicate in three independent experiments. Data shown are the mean ± SD of three biological replicates (**** *p* < 0.0001, two-way ANOVA, Tukey’s test); ns, not significant.

Based on these in vitro results, we then investigated the biological relevance of SUMO modification in cellular HIV reactivation across cell types. We repeated the inhibition experiments and assessed the viral reactivation in the Jurkat 3C9 and HC69 (human microglia) cells lines using a recently identified SUMO E2 inhibitor (2-D08) in vitro [66]. As shown in Figure 6C, 2-D08 significantly inhibited HIV reactivation in Jurkat 3C9 cells (*p* < 0.0001) in a dose-dependent manner. The same dose-dependent inhibition of HIV activation was not observed in the HC69 cell line. Taken together, these results suggest that the inhibition of SUMO modification reduces HIV reactivation in latently infected Jurkat cells, highlighting cellular tropisms of viral reactivation.

### 3.5. Protein–Protein Association (PPA) of Distinct Substrate Populations

Of the substrates identified by the proteomics dataset, 92 (1.81%) were present only in the unstimulated, latent HC69 cell population, and 78 (1.53%) were present only in the TNF-α-reactivated HC69 cell population. PPA analysis was performed for the substrates identified in the unstimulated (Figure S1A) and TNF-α-induced (Figure S1B) cell populations. Bioinformatic comparison of the substrates identified in the unstimulated, latent HIV population with other independent studies in different cell types using the Enrichr platform revealed forkhead L2 (FOXL2) as a major substrate to be previously described as a SUMO target [67,68,69]. SUMOylation of FOXL2 is essential for its stability, localization, and transcriptional repressive activity. FOXL2 is also highly enriched in HIV latently infected (GFP-) primary CD4+ T cells [70]. Tripartite motif-containing 24 (TRIM24) was also highly enriched in the latent HIV human HC69 and is a transcriptional regulator that interacts with PML nuclear bodies in a SUMO-dependent manner [71,72]. Importantly, TRIM24 functions to control HIV promoter activity, similar to other TRIM family proteins including TRIM28 and TRIM33 [73,74,75,76]. Sp3 transcription factor (SP3), a major substrate identified in the reactivated HIV human microglial HC69 model, has been previously linked to the host SUMOylation system.

**Figure 7:**
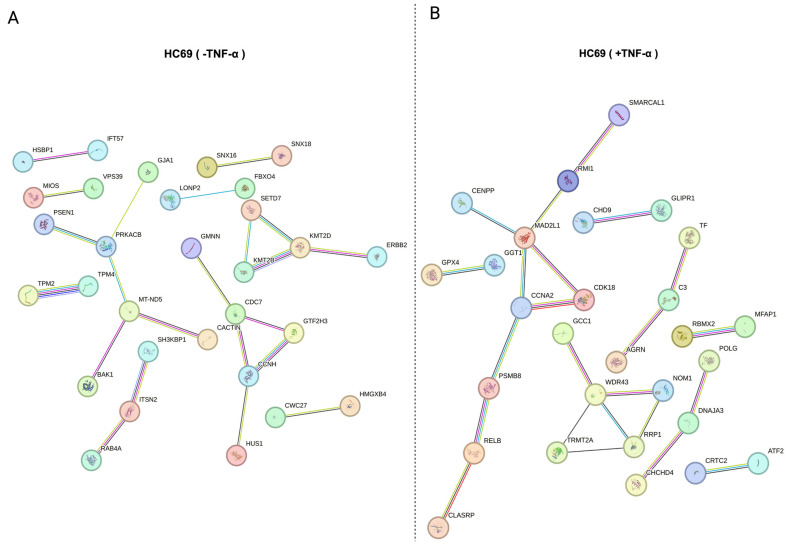
Protein–protein association (PPA) analysis of distinct protein populations. Interaction network of differentially expressed proteins present only in unstimulated (**A**) and TNF-α stimulated (**B**) HC69 microglia cells. Data were generated using the STRING database. The initial map of protein interactions from the STRING database, including the disconnected or isolated proteins without any protein–protein interactions and color legend, can be found in Figure S1.

Endogenous Sp3 is SUMOylated and localized to the nucleus and functions as a suppressor of LTR-driven transcription [77]. In a human microglial cell line, Sp1 transactivation of the HIV LTR is repressed by Sp3 [78]. Though Sp3 has been studied extensively as a SUMO substrate, it has not been previously authenticated as a SUMO substrate in HIV infection and viral latency. Gene ontology (GO) molecular function enrichment analysis of the search tool for the retrieval of interacting genes/proteins (STRING) identified unique protein interactions from HIV-latent and HIV-reactivated HC69 cells. Data showed that terms for the latent population included histone H3 methyltransferase and cAMP-dependent protein kinase activity and K48-linked polyubiquitin chains, the canonical signal for protein degradation (Figure 7A; Table 3). Enrichment analysis for latent HIV-reactivated populations included SH2 domain binding and exonuclease activity (Figure 7B; Table 4). These examples further support our proteomics dataset and are indicative of a relationship between HIV infection and SUMO modification.

## 4. Discussion

In this study, we further elucidated the intricate relationship between the host SUMOylation system and HIV infection. Our findings reveal a significant increase in SUMOylation within a human microglial latent model of HIV infection, HC69. This increased SUMOylation appears to be pivotal in maintaining the latency of the virus. However, SUMO conjugation is notably lost upon reactivation of microglia, underscoring the dynamic role of SUMOylation in HIV latency and reactivation. It is currently unknown which aspect of viral reactivation might induce this loss of SUMO conjugation, although our human frontal cortex immunofluorescence data suggest that the presence of HIV induces a loss of SUMO2/3. Our results are consistent with previous studies demonstrating that SUMOylation can influence various aspects of viral latency and reactivation. HIV takes advantage of the host ubiquitination and SUMOylation systems to modify its viral proteins, including p6 [62,79], IN [60,80], tat [81], and Nef [82], to achieve a productive infection. Additionally, HIV can redirect PTM systems to dictate the modification of host proteins. The HIV accessory protein Vpr seems to hijack the host ubiquitination system to target host anti-HIV factors for degradation [83]. Whether there are HIV accessory proteins capable of appropriating the host SUMOylation system to favor HIV-mediated SUMOylation of host proteins is unknown.

Our proteomics analysis revealed several key proteins with differential expressions between latent and reactivated cell lines, including ICAM1, histone H4, and components of the SMC5/6 complex. ICAM1′s increased expression in latent cells suggests a role in cellular adhesion and immune interactions that may support latency. Histone H4′s altered expression points to potential changes in chromatin structure and gene regulation. The differential expression of the SMC5/6 complex, involved in DNA repair and chromatin organization, highlights the role of SUMOylation in maintaining genomic stability and regulating latent HIV-1. We also found that HIV induces distinct proteomic profiles associated with HIV latency, or latency-associated proteins and reactivation in the HC69 cell line. The latent HIV HC69 cell line exhibited a distinct proteomic profile of proteins involved in cellular stress responses (TRIM24, FOXL2, PSEN1, and BAK1), chromatin remodeling (KMT2D, KMT2B, and SETD7), and immune modulation (CACTIN, TPM4, GJA1, and ITSN2). The presence of other latency-associated proteins, such as heat-shock proteins and chaperones, could indicate a cellular strategy to balance maintaining latency and managing stress responses that could potentially trigger viral reactivation. The proteins that were exclusively associated with the reactivated cell line were primarily associated with inflammatory responses, including signal transducers like NF-κB and transcription factors. The proteins associated with reactivation reflect how pro-inflammatory signals can overcome latency and highlight processes within the CNS that can lead to viral resurgence, potentially exacerbating neurological damage and contributing to neurocognitive disorders.

The observation that blocking the SUMOylation pathway reduces the percentage of GFP+ cells suggests that SUMOylation plays a critical role in the reactivation of latent HIV. Specifically, both TAK-981 and 2D-08 treatment effectively inhibit the reactivation process induced by TNFα, implicating the SUMOylation pathway as a potential therapeutic target for controlling HIV reactivation in T-cells. The lack of response to TAK-981 or 2-D08 in the HC69 microglial model suggests that there are other crucial factors that are involved in HIV reactivation in glial cells. To that end, by inhibiting SUMOylation, TAK-981 and 2-D08 likely disrupt the cellular machinery that supports viral reactivation, thus providing a promising avenue for therapeutic intervention. This finding is particularly relevant due to the challenge of eradicating latent HIV reservoirs.

Our study provides important insights into the role of SUMOylation in HIV latency and reactivation within microglia, highlighting its potential as a therapeutic target. By combining proteomics analysis with functional assays, we have elucidated key mechanisms underlying viral latency and identified potential targets for intervention. The data suggest that targeting the SUMOylation pathway could be a viable strategy to control viral reactivation and potentially reduce the size of the latent reservoir. The resources presented here will add a layer of insights into the extensive interplay between viral infections and the host SUMOylation system.

Elimination of reservoirs of latently infected cells is the greatest challenge to a comprehensive HIV cure. Latently infected cells are not easily distinguished, as the integrated virus remains transcriptionally silent and hidden from the host’s immune system. Novel strategies are necessary to identify and target latent viral reservoirs for immune-mediated clearance. Several in vitro and in vivo latency models have been established over the years, but latency reversal has remained elusive, and none are adequate models for understanding the contribution of glial cells as HIV reservoirs. Thus, novel research perspectives are needed to better understand the mechanisms of HIV persistence in glial cells. Our proteomics dataset can provide a platform for understanding the role of SUMOylation in HIV latency and exploring the therapeutic potential of SUMO inhibitors in the context of HIV infection, especially within the CNS. Although the work presented here explores a novel relationship between the host SUMOylation system in both glial and T cells, primary and animal models are necessary to provide a more efficient analysis of the latency-associated proteins identified in this study. The information from this study provides a great opportunity for the development of innovative and robust HIV latency models and further research on delineating the complexity of viral latency.

## Figures and Tables

**Table 1:** HIV auxiliary proteins and their major biological activity.

Protein	Major Function
Nef	Modulation of cellular receptors including CD4, CD28, and MHC I
Rev	Nuclear export of incompletely spliced viral mRNA
Tat	Transcriptional activation of HIV-1LTR
Vif	Stimulation of reverse transcription
Vpr	Nuclear import of pre-integration complex
Vpu	Promotes virion release through CD4 degradation in the ER

**Table 2:** Enrichr molecular function analysis for the most significantly enriched GO terms in the proteomic dataset.

Name	*p*-Value	Adjusted *p*-Value
Protein Phosphatase 2A Binding (GO: 0051721)	0.0007355	0.04096
NF-kappaB Binding (GO: 0051059)	0.0009417	0.04096
Small GTPase Binding (GO: 0031267)	0.005024	0.1110
Protein Homodimerization Activity (GO: 0042803)	0.005581	0.1110
GTPase Binding (GO: 0051020)	0.007053	0.1110
Cytochrome-B5 Redcutase Activity, Acting On NAD(P)H (GO: 0004128)	0.008720	0.1110
Interleukin-6 Receptor Binding (GO: 0005138)	0.01046	0.1110
NADH Dehydrogenase Activity (GO: 0003954)	0.01219	0.1110
Armadillo Repeat Domain Binding (GO: 0070016)	0.01219	0.1110
Protein Binding Involved In Heterotypic Cell-Cell Adhesion (GO: 0086080)	0.01564	0.1110

**Table 3:** Enrichr molecular function analysis for classification of substrates that change in expression during latent HIV-1 expression in HC69 cells, related to Figure 5A.

Name	*p*-Value	Adjusted *p*-Value
Histone H3 Methyltransferase Activity (GO: 0140938)	0.0006025	0.06901
Histone H3K4 Methyltransferase Activity (GO: 0042800)	0.001580	0.06901
Protein Heterodimerization Activity (GO: 0046982)	0.001851	0.06901
Protein-Lysine N-methyltransferase Activity (GO: 0016279)	0.001972	0.06901
Aspartic Endopeptidase Activity, intramembrane Cleaving (GO: 0042500)	0.02279	0.3049
cAMP-dependent Protein Kinase Activity (GO: 0004691)	0.02279	0.3049
Keratin Filament Binding (GO: 1990254)	0.02279	0.3049
Pyruvate Transmembrane Transporter Activity (GO: 0050833)	0.02279	0.3049
K48-linked Polyubiqutin Modification-Dependent Protein Binding (GO: 0036435)	0.02279	0.3049
Unmethylated CpG Binding (GO: 0045322)	0.03175	0.3049

**Table 4:** Enrichr molecular function analysis for classification of substrates that change in expression during TNF-α-induced reactivation of latent HIV-1 expression in HC69 cells, related to Figure 5B.

Name	*p*-Value	Adjusted *p*-Value
SH2 Domain Binding (GO: 0042169)	0.006489	0.1818
3′-5- Exonucease Activity (GO: 0008408)	0.01169	0.1818
Serine-Type Endopeptidase Inhibitor Activty (GO: 0004867)	0.01820	0.1818
Oligosaccharyl trasferase Activity (GO: 0004576)	0.01935	0.1818
Ceraminde 1-Phosphate Binding (GO: 1902387)	0.01935	0.1818
Ceramide 1-Phosphate Transfer Activity (GO: 1902388)	0.01935	0.1818
Protein Phosphatase 2B Binding (GO: 0030346)	0.01935	0.1818
Acid Phosphatase Activity (GO: 0003993)	0.02318	0.1818
Galactosidase Activity (GO: 0015925)	0.02318	0.1818
Beta-Galactosidase (CMP) Alpha-2,3-Sialytransferase Activity (GO: 0003836)	0.02318	0.1818

## Data Availability

The data presented in this study are available on request from the corresponding author.

## Supplementary Materials

The following supporting information can be downloaded at: https://www.mdpi.com/article/10.3390/cells14030235/s1, Figure S1: Protein–protein association (PPA) networks and key nodes in HC69 cells. PPA network constructed using STRING databases for (A) unstimulated (-TNF-α) and (B) stimulated (+TNF-α) HC69; Table S1: Functional enrichment analysis of the top 10 cellular components of key differentially expressed proteins; Table S2: Functional enrichment analysis of the top 10 molecular functions of key differentially expressed proteins; Table S3: Top 50 most differentially expressed proteins in HC69 -TNF-a vs. HC69 +TNF-a, as determined by the proteomics dataset obtained through mass spectrometry.
