# Supplementary material for: Comprehensive SUMO Proteomic Analyses Identify HIV Latency-Associated Proteins in Microglia
Source: Cells. 2025 Feb 6;14(3):235. doi: 10.3390/cells14030235 (PMC11817477; doi:10.3390/cells14030235)
Supplement: Supplementary file 1 [file cells-14-00235-s001.zip › cells-3352873-supplementary.pdf]

Table S1. Functional enrichment analysis of the top 10 cellular components of key differentially expressed proteins.

Index	Name	P-value	Adjusted p-value	Odds Ratio	Combined score
1	Intracellular Membrane-Bounded Organelle (GO:0043231)	6.176e-163	2.693e-160	2.61	974.22
2	Nucleus (GO:0005634)	1.122e-147	2.445e-145	2.58	874.05
3	Intracellular Non-Membrane-Bounded Organelle (GO:0043232)	1.698e-96	2.468e-94	3.59	791.18
4	Focal Adhesion (GO:0005925)	2.388e-82	2.603e-80	7.77	1459.86
5	Cell-Substrate Junction (GO:0030055)	1.251e-81	1.091e-79	7.51	1398.86
6	Nuclear Lumen (GO:0031981)	1.581e-71	1.149e-69	3.80	619.46
7	Nucleolus (GO:0005730)	3.029e-71	1.886e-69	3.82	619.80
8	Intracellular Organelle Lumen (GO:0070013)	1.057e-37	5.759e-36	2.53	215.69
9	U2-type Spliceosomal Complex (GO:0005684)	7.538e-33	3.652e-31	17.43	1289.49
10	Mitochondrial Matrix (GO:0005759)	2.438e-32	1.063e-30	3.64	264.72

Table S2. Functional enrichment analysis of the top 10 molecular functions of key differentially expressed proteins.

Index	Name	P-value	Adjusted p-value	Odds Ratio	Combined score
1	RNA Binding (GO:0003723)	9.247e-300	9.117e-297	8.43	5807.04
2	Cadherin Binding (GO:0045296)	7.345e-85	3.621e-82	10.41	2016.24
3	mRNA Binding (GO:0003729)	8.642e-49	2.840e-46	6.08	673.36
4	Purine Ribonucleoside Triphosphate Binding (GO:0035639)	2.761e-31	6.806e-29	3.02	212.53
5	Ubiquitin-Like Protein Ligase Binding (GO:0044389)	7.010e-22	1.382e-19	3.20	155.65
6	GTPase Binding (GO:0051020)	9.236e-22	1.518e-19	3.69	178.85
7	ATP Binding (GO:0005524)	9.220e-21	1.299e-18	3.16	145.58
8	Adenyl Ribonucleotide Binding (GO:0032559)	1.034e-19	1.274e-17	2.90	126.89
9	Ubiquitin Protein Ligase Binding (GO:0031625)	2.807e-19	3.076e-17	3.07	130.99
10	Small GTPase Binding (GO:0031267)	1.934e-17	1.907e-15	3.43	131.82

Table S3. Top 50 most differentially expressed proteins in HC69 -TNF-a vs. HC69 +TNF-a, as determined by the proteomics dataset obtained through mass spectrometry.

Symbol	Description	chr	logFC
ICAM1	intercellular adhesion molecule 1	19	4.9296
SOD2	superoxide dismutase 2	6	3.7576
PSMB10	proteasome 20S subunit beta 10	16	3.326
ENSA	endosulfine alpha	1	-3.1439
CNTNAP1	contactin associated protein 1	17	2.9932
ERAP1	endoplasmic reticulum aminopeptidase 1	5	2.868
NQO1	NAD(P)H quinone dehydrogenase 1	16	-2.8501
SMAP	NA	NA	-2.7984
THBS1	thrombospondin 1	15	-2.7782
TAPBP	TAP binding protein	6	2.7235
ITGA4	integrin subunit alpha 4	2	-2.6865
STRN3	striatin 3	14	2.6838
NFKBIA	NFKB inhibitor alpha	14	-2.6579
NFKB2	nuclear factor kappa B subunit 2	10	2.6464
CD82	CD82 molecule	11	2.6434
JPT1	Jupiter microtubule associated homolog 1	17	-2.5395
FGD4	FYVE, RhoGEF and PH domain containing 4	12	-2.4884
MAGT1	magnesium transporter 1	X	2.4323
CNPY4	canopy FGF signaling regulator 4	7	2.3931
TOP2A	DNA topoisomerase II alpha	17	-2.3324
PEX1	peroxisomal biogenesis factor 1	7	2.2764
TNFAIP2	TNF alpha induced protein 2	14	2.2736
HLA-C	major histocompatibility complex, class I, C	6	2.2682
CYGB	cytoglobin	17	-2.266
MGLL	monoglyceride lipase	3	2.2278
CDC42EP1	CDC42 effector protein 1	22	-2.2199
COL1A1	collagen type I alpha 1 chain	17	-2.2108
IFIT3	interferon induced protein with tetratricopeptide …	10	2.1717
TXN	thioredoxin	9	2.1079
PRR5L	proline rich 5 like	11	-2.0996
GBP2	guanylate binding protein 2	1	2.0945
BRMS1	BRMS1 transcriptional repressor and anoikis regula…	11	2.0803
DSG2	desmoglein 2	18	-2.0086
ARL2	ADP ribosylation factor like GTPase 2	11	-2.0078

TANC1	tetratricopeptide repeat, ankyrin repeat and coile...	2	-1.9728
SORBS3	sorbin and SH3 domain containing 3	8	-1.9532
NSA2	NSA2 ribosome biogenesis factor	5	1.9338
PSMB9	proteasome 20S subunit beta 9	6	1.9289
UXS1	UDP-glucuronate decarboxylase 1	2	1.8889
ANPEP	alanyl aminopeptidase, membrane	15	1.8592
ECE1	endothelin converting enzyme 1	1	1.8041
STOM	stomatin	9	-1.7959
NFKB1	nuclear factor kappa B subunit 1	4	1.7888
AKR1B1	aldo-keto reductase family 1 member B	7	1.7807
COMMD4	COMM domain containing 4	15	-1.7662
CA9	carbonic anhydrase 9	9	1.7584
RIPK2	receptor interacting serine/threonine kinase 2	8	1.744
ASS1	argininosuccinate synthase 1	9	1.7185
VIM	vimentin	10	1.7157

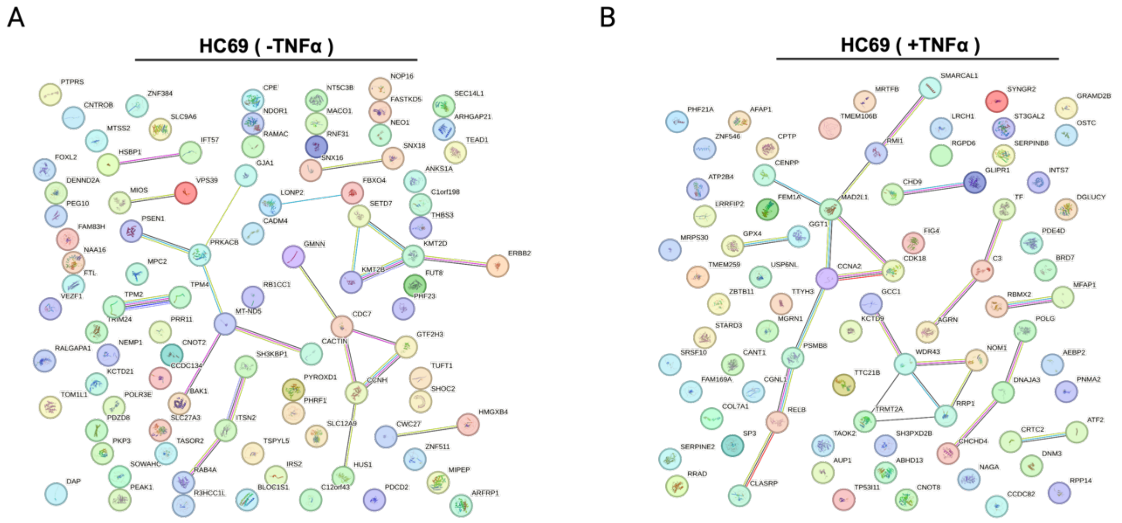

Figure S1. Protein-protein association (PPA) networks and key nodes in HC69 cells. PPA network constructed using STRING databases for (A) unstimulated (-TNFa) and (B) stimulated (+TNFa) HC69.
